# Exploring Speech Biosignatures for Traumatic Brain Injury and Neurodegeneration: Pilot Machine Learning Study

**DOI:** 10.2196/64624

**Published:** 2025-02-12

**Authors:** Rahmina Rubaiat, John Michael Templeton, Sandra L Schneider, Upeka De Silva, Samaneh Madanian, Christian Poellabauer

**Affiliations:** 1 Knight Foundation School of Computer and Information Sciences, Florida International University, Miami, FL, United States; 2 Department of Computer Science and Engineering, University of South Florida, Tampa, FL, United States; 3 Department of Communicative Sciences & Disorders, Saint Mary’s College, Notre Dame, IN, United States; 4 Department of Computer Science and Software Engineering, Auckland University of Technology, Auckland, New Zealand

**Keywords:** speech biosignatures, speech feature analysis, amyotrophic lateral sclerosis, ALS, neurodegenerative disease, Parkinson's disease, detection, speech, neurological, traumatic brain injury, concussion, mobile device, digital health, machine learning, mobile health, diagnosis, mobile phone

## Abstract

**Background:**

Speech features are increasingly linked to neurodegenerative and mental health conditions, offering the potential for early detection and differentiation between disorders. As interest in speech analysis grows, distinguishing between conditions becomes critical for reliable diagnosis and assessment.

**Objective:**

This pilot study explores speech biosignatures in two distinct neurodegenerative conditions: (1) mild traumatic brain injuries (eg, concussions) and (2) Parkinson disease (PD) as the neurodegenerative condition.

**Methods:**

The study included speech samples from 235 participants (97 concussed and 94 age-matched healthy controls, 29 PD and 15 healthy controls) for the PaTaKa test and 239 participants (91 concussed and 104 healthy controls, 29 PD and 15 healthy controls) for the Sustained Vowel (/ah/) test. Age-matched healthy controls were used. Young age-matched controls were used for concussion and respective age-matched controls for neurodegenerative participants (15 healthy samples for both tests). Data augmentation with noise was applied to balance small datasets for neurodegenerative and healthy controls. Machine learning models (support vector machine, decision tree, random forest, and Extreme Gradient Boosting) were employed using 37 temporal and spectral speech features. A 5-fold stratified cross-validation was used to evaluate classification performance.

**Results:**

For the PaTaKa test, classifiers performed well, achieving *F*_1_-scores above 0.9 for concussed versus healthy and concussed versus neurodegenerative classifications across all models. Initial tests using the original dataset for neurodegenerative versus healthy classification yielded very poor results, with *F*_1_-scores below 0.2 and accuracy under 30% (eg, below 12 out of 44 correctly classified samples) across all models. This underscored the need for data augmentation, which significantly improved performance to 60%‐70% (eg, 26‐31 out of 44 samples) accuracy. In contrast, the Sustained Vowel test showed mixed results; *F*_1_-scores remained high (more than 0.85 across all models) for concussed versus neurodegenerative classifications but were significantly lower for concussed versus healthy (0.59‐0.62) and neurodegenerative versus healthy (0.33‐0.77), depending on the model.

**Conclusions:**

This study highlights the potential of speech features as biomarkers for neurodegenerative conditions. The PaTaKa test exhibited strong discriminative ability, especially for concussed versus neurodegenerative and concussed versus healthy tasks, whereas challenges remain for neurodegenerative versus healthy classification. These findings emphasize the need for further exploration of speech-based tools for differential diagnosis and early identification in neurodegenerative health.

## Introduction

### Overview

The fields of health care and medical diagnostics have witnessed a significant shift toward noninvasive and accessible methods for early detection, assessment, and monitoring of medical conditions. This shift has been driven by technological advancements and growing research interest in digital health solutions [1]. Among these, speech analysis has emerged as a promising avenue, with studies identifying speech as a potential biosignature for a variety of neurodegenerative conditions [2,3]. The ability to reliably distinguish between conditions or detect coexisting disorders is critical for accurate diagnosis, tracking disease progression, and evaluating treatment effectiveness [4].

This pilot study investigates speech-based biosignatures of 2 distinct neurodegenerative conditions, that are, neurodegenerative diseases and mild traumatic brain injuries (mTBIs), specifically concussions. Speech patterns often reflect neurodegenerative health, with specific speech features showing promise for distinguishing between these conditions. The dataset includes individuals with concussions, patients with Parkinson disease (PD), and age-matched healthy controls for both groups (15 samples for each test). These groups were selected to ensure demographic compatibility while addressing the unique speech patterns associated with each condition.

Neurodegenerative diseases, such as PD, are characterized by the progressive loss of neurons in the brain and spinal cord, leading to impairments in motor and cognitive functions [5,6]. PD involves the degeneration of dopaminergic neurons, resulting in clinical symptoms such as tremors, rigidity, bradykinesia, and postural instability [7]. These symptoms worsen over time and lack curative treatments, necessitating reliable diagnostic tools for early intervention [8]. On the other hand, concussions, a form of mTBI, result from sudden trauma to the brain, causing temporary cognitive impairments, disruptions in brain function, and neurochemical changes. Repeated concussions are associated with a heightened risk of neurodegenerative disorders, such as dementia, later in life [9]. Despite their prevalence, approximately 90% of concussions go unreported, leading to inadequate medical attention and potentially catastrophic consequences [10].

Traditional diagnostic methods for neurodegenerative diseases and concussions often rely on observable motor symptoms, such as tremors, gait disturbances, or muscle rigidity, as well as subjective assessments of cognitive impairments [11]. However, emerging research has identified speech as a valuable biomarker for neurodegenerative health. Dysarthria and dysphonia, characterized by changes in articulation and motor speech production, are prevalent in both concussions and neurodegenerative conditions like PD [12-14]. Speech features, such as mel frequency cepstral coefficients (MFCCs), jitter, shimmer, harmonics-to-noise ratio (HNR), and other temporal and spectral attributes, have been shown to correlate with underlying neurodegenerative conditions.

In this study, we analyzed speech data from 2 well-established medical speech tasks, the PaTaKa task and the Sustained Vowel task. These tasks are widely used in clinical settings for assessing speech impairments. The objective of this study is to explore the potential of speech features in differentiating between concussions and neurodegenerative conditions, as well as their respective healthy controls, and to assess the feasibility of using these features as biomarkers for diagnosis. By addressing this objective, we aim to contribute to the development of speech-based diagnostic tools for early and accurate identification of neurodegenerative health conditions.

This study evaluated 37 speech-based features (25 temporal and 12 spectral), applying machine learning models such as support vector machine (SVM), decision tree (DT), random forest (RF), and Extreme Gradient Boosting (XGBoost) to classify between the groups.

The remainder of this paper describes our methodology, feature extraction and analysis, machine learning approaches, and results for the binary classification tasks across the 2 speech tests.

### Related Work

Diagnosing brain injuries and neurodegenerative diseases can be challenging; for instance, concussions may present subtle features that are difficult to detect, including using third person witness accounts of the injury, clinical examinations, and laboratory testing, where diagnostic accuracy is not always perfect [15]. Recent work has explored the diagnosis of concussions in athletes using mobile technologies [16] and speech analysis [17,18], while digital assessments, coupled with speech analysis, are also increasingly being used for individuals with neurodegenerative diseases [19]. In a study by Tsanas [19], various speech tasks have been used to distinguish between healthy people and individuals with PD, with relatively high accuracy. Other previous research has investigated the overall symptom severity of individuals with a neurodegenerative condition [11,20], the effectiveness of voice rehabilitation [21], and how to distinguish PD from other conditions such as essential tremor or atypical parkinsonism [22].

The choice of speech task is critical to obtaining speech samples that can be used for subsequent feature extraction and analysis. One commonly used speech task is to ask an individual to produce sustained phonation of vowels. For instance, the study by Mallela et al [23] presents an automatic voice assessment approach for separating healthy individuals from patients with amyotrophic lateral sclerosis (ALS). Although our study focuses exclusively on PD as the representative neurodegenerative condition, references to ALS studies are included to highlight the broader research landscape on neurodegenerative speech biosignatures and their diagnostic significance. Linear discriminant analysis is used to classify phonation, with the most successful model achieving more than 90% accuracy. Similarly, a study by Rueda and Krishnan [24] obtained sustained vowel data from 57 PD patients and 57 healthy individuals, and the study used 5 hierarchical and 1 partition-based clustering techniques to compare and cross-check PD patients at different phases. In some cases, researchers have relied on existing voice recordings, for example, obtained through the Parkinson’s Voice Initiative project (the largest speech-PD dataset so far) to analyze voice impairment due to PD [25].

Daudet et al [18] developed a mobile app to diagnose concussions, using data from 47 high-schools and colleges in the Midwest. The study used several speech tasks such as repetition of a sequential motion rate, alternating motion rate, multisyllabic words (words with 4 syllables containing front, middle, and back vowels, and bilabial, alveolar, velar, and glide consonants). The work by Vashkevich et al [26] presented features for detecting pathological changes in acoustic speech signals for ALS diagnosis. It used recordings from 48 people (26 with ALS) and investigated vowel harmony. The features obtained an 88% correct classification performance using linear discriminant analysis. Various speech-based indicators, such as shimmer, jitter, HNR, and other temporal and spectral indicators, have also been explored as dysphonia measures in individuals with neurodegenerative diseases [27]. Finally, in a study by Benba et al [22], the authors investigated the most effective acoustic elements for accurately identifying symptoms of PD, combining shimmer, jitter, pitch, harmonicity, pulses, and voicing by using K-Nearest Neighbor classifiers with different types of kernels (ie, radial basis functions, linear, polynomial, and multilayer perceptron).

Machine learning–based solutions have become the standard for most health care decision-making processes, for example, most previous works focus on differentiating diseased individuals from healthy controls. For example, the work by Tsanas and Arora [28] evaluated 2289 individuals (2023 healthy controls and 246 PD patients) and analyzed 15,227 voice tasks (9994 for healthy controls and 5233 for PD patients). Similarly, the work Bongioanni [29] compared speech-based automatic classification of patients with ALS and healthy people using sustained phoneme generation, diadochokinetic task, and spontaneous speech. They classified voice samples from 25 patients with ALS and 25 healthy participants using SVMs and deep neural networks. More recently, more focus has been given to multiclass scenarios, for example, the study by Benba et al [22] used a Convolutional Neural Network Long Short-term Memory to categorize ALS, PD, and healthy controls. The study analyzed speech data from 60 people, focusing on sentence reading, sound repetition, and sustained vowels.

Though there are studies that had investigate speech features pertaining to neurodegenerative disorders or acquired neurodegenerative disorders like mTBI, there are not many studies exploring speech feature variations between those populations which might co-occur and impact speech production differently.

The aim of this study is to investigate whether distinct speech-based biomarkers, derived from commonly used tasks like the PaTaKa and Sustained Vowel tests, can effectively differentiate between concussed individuals, neurodegenerative conditions (focused on PD), and healthy controls.

## Methods

### Data Collection

This study focused on 2 widely used speech tasks, the sequential motion rate task (PaTaKa test) and the Sustained Vowel test. The PaTaKa test evaluates speech-motor function by asking participants to take a deep breath and repeatedly articulate “Pa-Ta-Ka” as steadily as possible in 1 breath, providing insights into the rate and precision of sequential articulatory actions.

In the Sustained Vowel test, participants were instructed to sustain the vowel sound “ah” for as long as possible, offering valuable information about voice quality and potential vocal tremor. Both tasks were assigned to four participant groups, that are (1) individuals with concussions, (2) individuals with neurodegenerative conditions (specifically PD), (3) healthy controls age-matched to the concussed group, and (4) healthy controls age-matched to the neurodegenerative group.

Individuals diagnosed with a concussion were evaluated by physicians or athletic trainers using standardized neurocognitive assessment tools, such as ImPACT (Immediate Post-Concussion Assessment and Cognitive Testing) by ImPACT Applications, Inc, SCAT (Sport Concussion Assessment Tool), an open-access tool , and SAC (Standardized Assessment of Concussion) by researchers at the University of North Carolina’s Sports Medicine Research Laboratory, within 48 hours of the suspected injury. Individuals with neurodegenerative conditions (ie, PD) were diagnosed by licensed neurologists or family physicians. All participants with PD were in the early stages of disease progression (Hoehn and Yahr stage 1‐2) and were assessed using tools such as the MDS-UPDRS (Movement Disorder Society - Unified Parkinson’s Disease Rating Scale) and Hoehn and Yahr Scale.

Healthy controls were divided into two groups: (1) young healthy individuals age-matched to the concussed group and (2) older healthy individuals age-matched to the neurodegenerative group. This separation ensures more accurate comparisons between the groups, minimizing the confounding effects of age-related speech differences.

Participants completed the speech tasks using a mobile app (smartphone or tablet) that provided both visual and auditory instructions. The app also recorded the audio samples digitally for subsequent analysis. Audio data were collected from a total of 235 and 239 participants for the PaTaKa and Sustained Vowel tests, respectively, as shown in Table 1.

**Table 1.** Description of collected samples.

Test name and population		Samples, n	Sex		Age (years), mean (SD)
			Male, n	Female, n	
PaTaKa					
	Concussed	97	86	11	17 (3)
	Healthy control (young)	94	81	13	17 (3)
	Neurodegenerative (PD^a^)	29	17	12	63.67 (4.95)
	Healthy control (older)	15	5	10	63.67 (4.95)
Sustained Vowel					
	Concussed	91	82	9	17 (3)
	Healthy control (young)	104	90	14	17 (3)
	Neurodegenerative (PD)	29	17	12	63.67 (4.95)
	Healthy control (older)	15	5	10	63.67 (4.95)

a PD: Parkinson disease.

The PaTaKa test dataset includes speech samples from 97 concussed participants, 29 participants with neurodegenerative conditions (ie, PD), 97 age-matched young healthy controls, and 15 age-matched older healthy controls. Similarly, the Sustained Vowel dataset consists of speech samples from 91 concussed participants, 29 participants with neurodegenerative conditions (ie, PD), 91 age-matched young healthy controls, and 15 age-matched older healthy controls.

**Figure 1.**
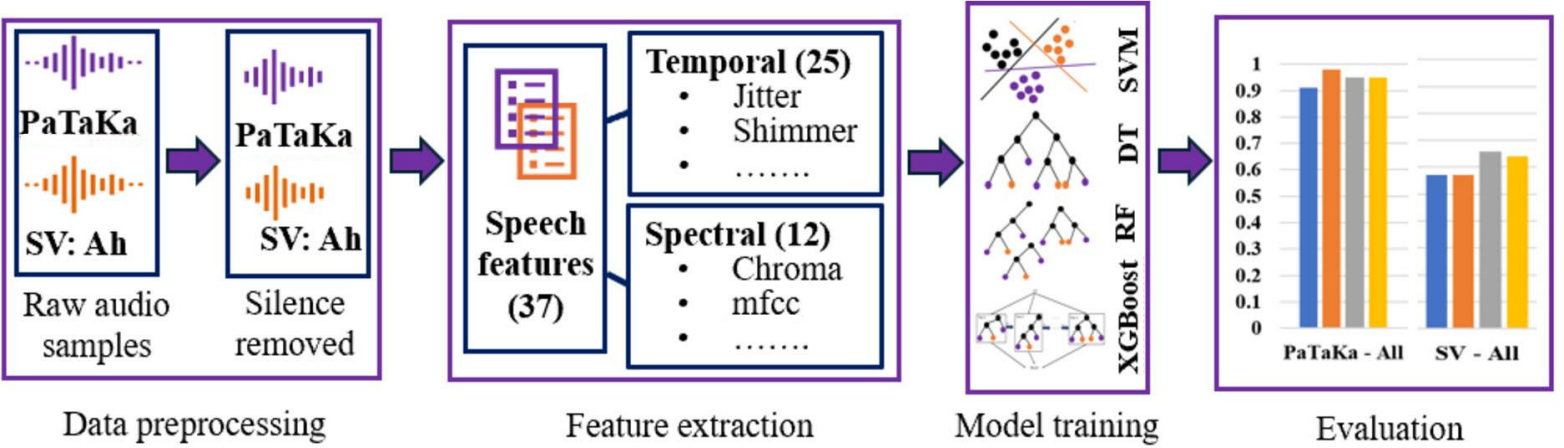
Overall visualization of the 4 methodological steps: data preprocessing, feature extraction, model training, evaluation. DT: decision tree; mfcc: mel frequency cepstral coefficient; RF: random forest; SV: Sustained Vowel; SVM: support vector machine; XGBoost: Extreme Gradient Boosting.

In the remainder of this section, we describe the 4 key components of the proposed analysis methodology, shown in Figure 1, that are data preprocessing, feature extraction, model training, and evaluation.

### Data Preprocessing

The voiced portions of speech signals typically carry the most critical information for analysis. Therefore, to enhance the quality and efficiency of feature extraction, it is essential to eliminate unnecessary components, such as silence intervals and extraneous noise, during the preprocessing phase. In this study, silence intervals were removed at 2 points in each speech recording using the free software developed by Muse group named “Audacity”. Specifically, silence was cut from the beginning of the recording to the onset of vocalization and from the offset of vocalization to the end of the recording.

In addition, recordings that did not meet the study’s requirements, such as those where participants failed to produce the expected utterances (eg, “PaTaKa” in 1 continuous breath or sustained vowel production without interruptions), were excluded from further analysis. This step ensured a high-quality dataset for feature extraction and classification, thereby improving the reliability of the results.

### Data Augmentation

To address the challenges of imbalanced datasets and improve classification performance, data augmentation was applied to specific data subsets, particularly those with limited samples, such as the neurodegenerative (ie, PD) and age-matched healthy datasets. The augmentation process involved adding Gaussian noise to the raw audio signals. The noise factor was set to 0.005 to ensure that the original speech characteristics were preserved while introducing subtle variations to increase sample diversity. For each audio file, a noise vector was generated using a Gaussian distribution, scaled by the specified noise factor, and added to the original signal. The augmented audio signals were then normalized to ensure they remained within the acceptable amplitude range for further processing.

This step increased the dataset size from 29 PD and 15 healthy samples to 58 PD and 30 healthy samples, resulting in a notable improvement in classification accuracy from under 30% (original data) to 60%‐70% (augmented data).

### Feature Extraction

Feature extraction is the process of transforming raw audio data into numerical features while retaining the critical information embedded within the original signal. Among various methods for converting speech into numerical data, temporal and spectral features are widely used in speech-processing research [22,26,27,30]. In these studies, both types of features were extracted using Python’s Librosa library [31].

Temporal features describe the changes in an audio signal over time, such as amplitude and pitch variation. This study extracted 25 temporal features, including 4 fundamental frequency measures (eg, mean and SD of F0), 5 jitter measures, 6 shimmer measures, and the HNR. These features provide insights into voice quality and stability, commonly associated with motor speech dysfunctions. The full list and descriptions of these temporal features are provided in Multimedia Appendix 1.

Spectral features analyze the frequency components of the speech signal and are commonly used in applications such as speech recognition and speaker identification. This study extracted 12 spectral features, including MFCC, spectral centroid, chroma features, and spectral flatness. These features capture frequency-domain characteristics that are sensitive to articulation and vocal tract configurations. Detailed descriptions of these spectral features are presented in Multimedia Appendix 1.

All 37 extracted features (25 temporal and 12 spectral) were included in the training and evaluation of machine learning models. By retaining the full feature set, we ensured that potentially valuable information was preserved, particularly given the small sample size. Data augmentation techniques, such as adding noise to the audio samples, were used to improve the robustness of the models and enhance performance, especially for the classification between neurodegenerative and healthy controls, where the original dataset resulted in poor classification performance.

### Model Training

In recent years, the trend in digital health care has been to use machine learning models to classify input data (speech samples) into 2 or more classes based on extracted features. In this work, we employed several popular machine learning techniques, such as SVM, DT, RF, and XGBoost [18]. These models were chosen due to their interpretability, robustness, and ability to handle small datasets effectively, which is essential for clinical applications.

SVM, a supervised learning algorithm proposed by Boser et al [32], is grounded in statistical learning theory and is particularly effective for high-dimensional data [33]. It uses hyperplanes and margins to separate data into classes, with its performance being highly dependent on data scaling and the choice of kernel functions. DTs, on the other hand, divide feature space into regions by recursively splitting data and assigning classes to leaf nodes [34]. Despite their simplicity, DTs are prone to overfitting, especially on small datasets.

RFs mitigate this issue by employing an ensemble of DTs trained on bootstrapped datasets, with each tree built using a random subset of features [35]. The final class prediction is based on a majority vote across all trees, which reduces variance and enhances model robustness. Finally, XGBoost, a gradient boosting implementation, constructs DTs sequentially, optimizing performance by correcting errors from previous iterations [36]. It is known for its computational efficiency and scalability, making it a popular choice for structured datasets. For a given sample, the final prediction can be calculated by summing up the scores of overall leaves, which is illustrated in Multimedia Appendix 2.

Given the limited size of our dataset, we prioritized traditional machine-learning models over deep learning methods. While deep learning algorithms have demonstrated exceptional performance on large datasets, their effectiveness diminishes with smaller datasets due to overfitting and computational requirements. Traditional machine learning models, such as SVM and RF, offer superior interpretability, which is critical for clinical decision-making [28]. For instance, the study by Pishgar et al [37] found that on a small voice disorder dataset, SVM outperformed a deep neural network in terms of sensitivity and specificity.

In this study, all 37 extracted features (25 temporal and 12 spectral) were used without any feature selection or filtering. Data augmentation was applied to address the limited sample size, particularly for the neurodegenerative versus healthy dataset, where the augmented dataset improved model performance.

To train and evaluate the machine learning models, we applied a 75‐25 stratified split of the dataset into training and test sets, ensuring that class distributions were preserved. Stratified 5-fold cross-validation was used to evaluate model performance more reliably, and Grid Search was used to fine-tune hyperparameters for all algorithms.

### Evaluation

In this study, we assessed the performance of our classification models using multiple evaluation metrics, with a particular focus on the *F*_1_-score due to its robustness in handling unbalanced datasets. The *F*_1_-score is particularly well-suited for situations where there is an imbalance in the class distribution, as it provides a harmonic mean of precision and recall, balancing the trade-off between these 2 metrics. The *F*_1_-score is defined as follows in Multimedia Appendix 2.

Both precision and recall are crucial in medical applications, where the consequences of false positives or false negatives can be severe. The *F*_1_-score offers a balanced view of a model’s performance when neither precision nor recall can be prioritized over the other. A higher *F*_1_-score (ranging from 0 to 1) indicates a better-performing model.

In addition to the *F*_1_-score, we evaluated our models using precision, recall, and accuracy to provide a comprehensive view of model performance. These metrics helped compare the performance of models across different speech tasks (PaTaKa and Sustained Vowel) and combinations (eg, concussed vs healthy, concussed vs neurodegenerative, neurodegenerative vs healthy). The results section discusses these findings in detail, highlighting the implications of our model’s performance for clinical applications.

### Ethical Considerations

This research was conducted in compliance with ethical standards and approved by the Institutional Review Board at the University of Notre Dame. The approval numbers for this study are 18-01-4338 and 18-01-4340 for PD and concussion, respectively. All participants provided informed consent (MultimediaAppendices 3  4), and their confidentiality was ensured throughout the study.

## Results

### Overview

**Figure 2.**
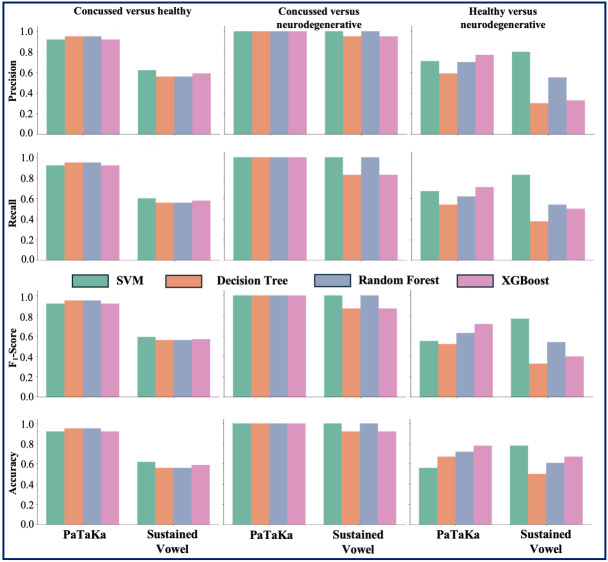
Performance metrics by test type, model, and combination. SVM: support vector machine; XGBoost: Extreme Gradient Boosting.

The performance of the models was evaluated using precision, recall, *F*_1_-score, and accuracy across 3 participant combinations (ie, concussed vs healthy, concussed vs neurodegenerative, and neurodegenerative vs healthy) for 2 widely used speech tasks, PaTaKa and Sustained Vowel. The results provide insights into the discriminative ability of each test and highlight the comparative effectiveness of different classifiers in distinguishing between participant groups. While the PaTaKa task generally demonstrated robust performance across all combinations, the Sustained Vowel test showed varying levels of accuracy, particularly for certain groups and classifiers. The performance for each combination and test, along with discussions on their implications are illustrated in Figure 2.

### Concussed Versus Healthy

#### PaTaKa Test

The models performed exceptionally well, achieving near-perfect precision, recall, *F*_1_-score, and accuracy across all classifiers. DT and RF slightly outperformed SVM and XGBoost, consistently achieving 0.95. There are no sources in the current document across all metrics. These results highlight the PaTaKa test’s robustness in distinguishing between concussed and healthy participants.

#### Sustained Vowel Test

Performance dropped significantly compared with the PaTaKa test. SVM and XGBoost achieved slightly higher metrics, with *F*_1_-scores around 0.59‐0.62. DT and RF had the lowest performance, with metrics around 0.56. The reduced performance might indicate that sustained vowels are less effective for distinguishing concussed participants from healthy individuals.

### Concussed Versus Neurodegenerative

#### PaTaKa Test

All models performed perfectly, achieving precision, recall, *F*_1_-score, and accuracy of 1.0. This demonstrates the effectiveness of the PaTaKa test for differentiating concussed participants from those with neurodegenerative conditions. Consistency across all classifiers reinforces the reliability of this task for this combination.

#### Sustained Vowel Test

Similar to the PaTaKa test, most models achieved perfect scores across all metrics. However, DT and XGBoost showed slightly reduced performance, with *F*_1_-scores of 0.87 and accuracy of 0.92. Despite slight variability, the Sustained Vowel test remains a strong indicator for distinguishing these groups.

### Neurodegenerative Versus Healthy

#### PaTaKa Test

Results varied significantly across classifiers. RF and XGBoost outperformed others, achieving *F*_1_-scores of 0.63 and 0.72, respectively. DT and SVM performed poorly, with *F*_1_-scores around 0.52‐0.55. These results indicate that the PaTaKa test has moderate effectiveness for this group but requires careful classifier selection.

#### Sustained Vowel Test

Similar trends were observed. XGBoost achieved the highest *F*_1_-score (0.40) and accuracy (0.67), while other models showed significantly lower performance. This underscores the challenge of distinguishing neurodegenerative participants from healthy controls using sustained vowel tasks.

### Feature Set Analysis

Understanding the importance of individual features in classification tasks is crucial for interpreting the predictive power of machine learning models. In this study, we examined feature importance across all tests and combinations to identify the most influential speech features contributing to the classification of concussed, neurodegenerative, and healthy individuals. Feature importance was calculated for each model (SVM, DT, RF, and XGBoost) using a combination of metrics, such as Gini importance, SHAP values, or permutation importance, depending on the model.

To identify globally significant features, we analyzed the frequency of features ranked among the top 5 across all 24 tests. A summary of the top 10 most frequent features is presented in Table 2, while Table 3 provides combination-specific feature importance values. The most frequently identified features were temporal and spectral characteristics, which are known to capture both short-term and long-term speech patterns.

**Table 2.** Top 10 most frequent features across all tests.

Rank	Feature	Frequency	Mean importance
1	duration	15	0.29
2	zero_crossing_rate	13	0.33
3	spectral_flatness	12	0.30
4	mfcc^a^	11	0.25
5	spectral_bandwidth	7	0.42
6	spectral_centroid	6	0.07
7	spectral_contrast	5	0.07
8	chroma_stft	5	0.32
9	HNR^b^	4	0.06
10	f4_median	4	0.04

a mfcc: mel frequency cepstral coefficient.

b HNR: harmonics-to-noise ratio.

**Table 3.** Combination specific feature importance value.

Combination and test		Feature	Value
**Concussed versus healthy**			
	PaTaKa	Duration	1.9
	PaTaKa	Zero-crossing rate	0.47
	Sustained Vowel	Spectral flatness	0.12
**Concussed versus neurodegenerative**			
	PaTaKa	Spectral bandwidth	1.3
	Sustained Vowel	MFCC^a^	2.9
**Neurodegenerative versus healthy**			
	PaTaKa	HNR^b^	0.43
	Sustained Vowel	Spectral flatness	0.76

a MFCC: mel frequency cepstral coefficient.

b HNR: harmonics-to-noise ratio.

Among the top 10 features, duration, zero-crossing rate, and spectral_flatness were the most influential, appearing consistently across multiple tests and combinations. These features reflect critical aspects of speech production, including articulation rate, periodicity, and frequency smoothness. For instance:

- Duration: This feature provides insights into motor control and speech articulation by measuring the length of utterances.
- Zero-crossing rate: Indicative of voice signal periodicity, this feature is particularly significant in distinguishing voiced and unvoiced speech segments.
- Spectral_flatness: This feature quantifies the uniformity of the speech spectrum, distinguishing between harmonic and noise-like components.

Combination-specific patterns further highlight the variability in feature importance depending on the test (PaTaKa or Sustained Vowel) and the target classification task (concussed vs healthy, concussed vs neurodegenerative, and neurodegenerative vs healthy). For example, (1) in the concussed versus healthy classification, features like mfcc and spectral bandwidth were highly impactful, particularly in the PaTaKa test, (2) in the Concussed concussed versus neurodegenerative classification, spectral_centroid and chroma_stft played a significant role in distinguishing between the 2 groups, and (3) for the neurodegenerative versus healthy classification, features such as f4_median and HNR were key discriminators, particularly in the Sustained Vowel test.

**Figure 3.**
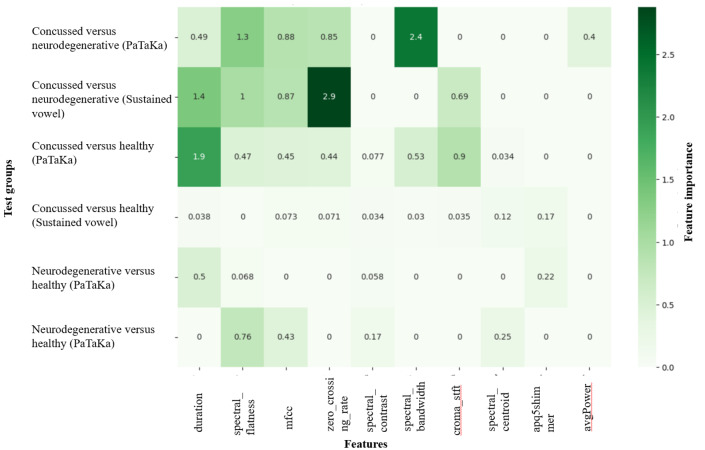
Top 10 most frequent features across all tests. mfcc: mel frequency cepstral coefficients.

The distribution of feature importance values across combinations and tests is visualized in Figure 3, while the detailed numerical values for each combination and test are available in Table 3. These findings emphasize the variability of feature contributions across different tasks and highlight the importance of task-specific feature analysis for robust classification.

## Discussion

### Principal Findings

The findings of this study provide valuable insights into the use of speech-based features for differentiating between neurodegenerative conditions, particularly mTBI (concussions) and neurodegenerative diseases (eg, PD). By leveraging 2 commonly used speech tasks, the PaTaKa test and the Sustained Vowel test, and a variety of machine learning models, we achieved classification accuracies ranging from 60% to 90%, with RF and XGBoost models consistently outperforming others. In addition, we identified key speech features, such as duration, zero-crossing rate, and spectral flatness, as critical biomarkers for distinguishing between these conditions. These results underscore the potential of speech features as noninvasive biomarkers for neurodegenerative health assessment and highlight the complementary roles of the PaTaKa and Sustained Vowel tests in revealing task-specific and globally significant features.

### Key Observations

First, task-specific performance. The PaTaKa test consistently outperformed the Sustained Vowel test across all combinations. This may be attributed to the sequential articulatory movements required in the PaTaKa test, which can better capture subtle motor and speech deficits. For example, in the concussed versus healthy classification, *F*_1_-scores for PaTaKa exceeded 0.9 across all models, whereas the Sustained Vowel test achieved *F*_1_-scores below 0.6 for the same classification. These findings highlight the importance of task selection in speech analysis and suggest that diadochokinetic tasks may provide richer diagnostic information.

Second, model-specific trends. Among the machine learning models, RF and XGBoost consistently performed well, demonstrating their ability to handle complex, nonlinear relationships in speech data. This aligns with previous research highlighting the robustness of ensemble learning methods in biomedical and speech signal processing tasks [38].

Third, the high interpretability of DTs also provides an advantage for clinical applications, particularly in scenarios where transparency is critical for adoption in health care settings.

Fourth, despite its slightly lower performance in some scenarios, DT models remain valuable due to their simplicity and ease of implementation.

Fifth, interestingly, SVMs displayed strong performance in balanced datasets, particularly in the concussed versus neurodegenerative classification, where precision and recall consistently reached 1.0 for the PaTaKa test. This finding is consistent with previous studies showing that SVMs are effective for high-dimensional data, especially when datasets are carefully preprocessed and balanced [39]. The performance of SVM in this classification task further underscores its utility in distinguishing nuanced differences between distinct neurodegenerative conditions using speech features.

Finally, feature importance. The analysis of feature importance revealed that a small subset of features consistently played a dominant role across tests and combinations. Temporal features such as duration and zero-crossing rate were particularly influential, likely reflecting disruptions in motor control and speech rhythm caused by both concussions and neurodegenerative conditions. Spectral features, including spectral_flatness, mfcc, and spectral_bandwidth, were also critical, highlighting their utility in capturing frequency-domain variations associated with speech pathologies. These results align with previous research, which has emphasized the role of both temporal and spectral features in detecting neurodegenerative impairments.

### Comparison With Previous Studies

Our findings corroborate and extend existing literature on speech-based biomarkers for neurodegenerative conditions. Previous research has demonstrated the utility of features such as MFCC and jitter for detecting PD [4], as well as features like zero-crossing rate and duration for identifying concussions [19]. However, this study uniquely emphasizes the differentiation between neurodegenerative diseases like PD and mild traumatic brain injury (eg, concussions), a task that remains relatively underexplored in existing literature.

Furthermore, the inclusion of both PaTaKa and Sustained Vowel tests enables a more comprehensive analysis of task-specific feature relevance. While previous studies have evaluated the diagnostic utility of individual speech tasks (eg, sustained phonation for ALS in studies by Allison et al [13] and Tsanas et al [27]), this work highlights how combining multiple tasks can reveal unique and complementary insights into speech biosignatures associated with diverse neurodegenerative conditions.

In addition to confirming the significance of widely used features such as spectral flatness and zero-crossing rate, our study identifies new combinations of features, including spectral contrast and chroma-based features, as being critical for distinguishing between these groups. These results align with recent advancements in the field, where ensemble learning models, such as RF and XGBoost, are increasingly used to capture the intricate, nonlinear relationships within speech data [23].

By addressing age-related variability and introducing data augmentation to mitigate the challenges of limited datasets, this study not only validates previously established findings but also sets the stage for future research aimed at improving the diagnostic accuracy of speech-based assessments across distinct but potentially overlapping neurodegenerative conditions.

### Implications for Clinical Practice

The results of this study highlight several practical implications for clinical applications.

First, noninvasive diagnostics. The reliance on speech features, which can be collected using readily available devices like smartphones, opens up possibilities for remote and noninvasive diagnostics. This is particularly valuable in resource-constrained settings where access to advanced imaging or neurophysiological tests may be limited.

Second, early detection. The ability to detect subtle speech impairments associated with neurodegenerative conditions could enable earlier diagnosis, allowing for timely interventions.

Finally, task selection. The superior performance of the PaTaKa test suggests that it should be prioritized in future speech-based diagnostic protocols, particularly for distinguishing between concussions and neurodegenerative conditions.

### Limitations

Despite the promising results, there are several limitations to this study.

First, small dataset—the dataset size, particularly for neurodegenerative diseases, was relatively small. This may limit the generalizability of the findings to larger, more diverse populations.

Second, demographic differences—the age gap between the concussed (younger) and neurodegenerative (older) populations poses a potential confounding factor. While age-matched healthy controls were included, the results could be influenced by inherent age-related differences in speech production.

Third, feature engineering and contextual factors—while the study identified important features, the reliance on manual feature extraction may overlook nuanced patterns. Advanced techniques, such as deep learning–based feature discovery, could reveal hidden characteristics in speech data. Future research should also account for comorbidities and age-related factors, as these can influence speech biosignatures and potentially confound results. Age-normalized datasets and statistical adjustments can further enhance the robustness of classification models.

### Future Directions

This study demonstrates the potential of speech-based features to differentiate between concussed, neurodegenerative, and healthy individuals. While promising, the findings also highlight several areas for improvement and expansion, which we aim to address in future work.

First, dataset expansion and diversity. The current dataset includes limited samples from each group, particularly for neurodegenerative diseases. Future studies will expand the dataset to include larger and more diverse populations, ensuring broader generalizability of the results. In addition, we aim to achieve a more balanced age distribution across all participant groups, enabling more robust analyses and minimizing potential biases.

Second, age-related effects. While we mitigated some confounding effects of age by including 2 distinct healthy control groups (age-matched for concussed and neurodegenerative participants), future studies will incorporate more advanced strategies to address age-related variations in speech features. These include (1) explicitly including age as a covariate in statistical models to control its effects and quantify its influence on the results, (2) conducting age-matched subgroup analyses to validate that classification performance is not driven by age-related biases but by the underlying neurodegenerative conditions, and (3) expanding the dataset to improve the representation of younger and older age groups across all conditions.

Third, feature engineering and discovery. While this study focused on predefined temporal and spectral features, advanced deep learning models such as autoencoders or transformer-based models could uncover latent features that may better distinguish between neurodegenerative conditions. In addition, further exploration of task-specific feature relevance could reveal complementary insights into speech patterns for different health conditions.

Fourth, longitudinal data analysis. Future work should explore longitudinal data to track changes in speech biosignatures over time. This would help identify temporal patterns associated with disease progression and recovery, providing valuable insights for monitoring treatment efficacy and early diagnosis.

Fifth, integration with clinical practice. To enhance the clinical utility of this research, future efforts should focus on integrating speech-based diagnostic tools into real-world health care settings. This includes (1) developing user-friendly mobile apps or web applications for noninvasive speech analysis and (2) collaborating with clinicians to validate the models and evaluate their effectiveness in clinical decision making processes.

Finally, evaluation metrics and benchmarking. Expanding the evaluation metrics to include area under the receiver operating characteristic curve and precision-recall curves would provide a more comprehensive understanding of model performance. In addition, benchmarking against existing speech-based models or alternative diagnostic tools could further contextualize the findings and demonstrate the added value of the proposed methods.

By addressing these areas, future research can build upon the findings of this study to further advance the field of speech analysis in neurodegenerative health, improve diagnostic accuracy, and pave the way for noninvasive, scalable diagnostic tools.

### Conclusion

This study demonstrates the potential of speech features, particularly those derived from the PaTaKa test, as effective biomarkers for distinguishing between concussed, neurodegenerative, and healthy individuals. By identifying task-specific and globally important features, the findings lay the groundwork for developing noninvasive, speech-based diagnostic tools that can be readily implemented in clinical practice. Further research addressing the study’s limitations could pave the way for broader applications of speech analysis in neurodegenerative health.

## Supplementary material

10.2196/64624 Multimedia Appendix 1 Feature description.

10.2196/64624 Multimedia Appendix 2 Equations.

10.2196/64624 Multimedia Appendix 3 Consent form for participants with neurodegenerative conditions.

10.2196/64624 Multimedia Appendix 4 Consent form for participants with concussions.

## Abbreviations

ALS: amyotrophic lateral sclerosis
DT: decision tree
HNR: harmonics-to-noise ratio
ImPACT: Immediate Post-Concussion Assessment and Cognitive Testing
MDS-UPDRS: Movement Disorder Society - Unified Parkinson's Disease Rating Scale
MFCC: mel frequency cepstral coefficient
mTBI: mild traumatic brain injury
PD: Parkinson disease
RF: random forest
SAC: Standardized Assessment of Concussion
SCAT: Sport Concussion Assessment Tool
SVM: support vector machine
XGBoost: Extreme Gradient Boosting
