# Supplementary material for: Exploring Speech Biosignatures for Traumatic Brain Injury and Neurodegeneration: Pilot Machine Learning Study
Source: JMIR Neurotechnol. 2025 Feb 12;4:e64624. doi: 10.2196/64624 (PMC12671332; doi:10.2196/64624)
Supplement: Multimedia Appendix 1 [file neuro-v4-e64624-s001.docx]

Table 1: **Description of temporal features.**

| **Feature** | **Description** |
| --- | --- |
| Duration | Length of the audio recording for an individual to complete the task. |
| meanF0Hz | Mean value of fundamental frequency F0. |
| stdevF0Hz | Standard Deviation of fundamental frequency F0. |
| HNR | Harmonics-to-Noise ratio. |
| localabsoluteJitter | Fundamental Frequency Perturbation (absolute) rapJitter. |
| RAP | Relative Amplitude Perturbation. |
| ppq5Jitter | Five-point Period Perturbation Quotient. |
| ddpJitter | Average absolute difference of differences between cycles, divided by the average period. |
| localShimmer | Local Amplitude Perturbation. |
| localdbShimmer | Local Amplitude Perturbation (decibels). |
| apq3Shimmer | Three-point Amplitude Perturbation Quotient. |
| apq5Shimmer | Five-point Amplitude Perturbation Quotient. |
| apq11Shimmer | Eleven-point Amplitude Perturbation Quotient. |
| ddaShimmer | Average absolute difference between consecutive differences between amplitudes of consecutive periods. |
| crossing rate | The rate at which a signal changes from positive to negative or from negative to positive. |
| AvgPower | Average amount of work done or energy converted per unit of time. |
| f1_mean | Average frequency of the first formant in a speech signal. |
| f2_mean | Average frequency of the second formant in a speech signal. |
| f3_mean | Average frequency of the third formant in a speech signal. |
| f4_mean | Average frequency of the fourth formant in a speech signal. |
| f1_median | Measure the frequency of the first formant. |
| f2_median | Measure the frequency of the second formant. |
| f3_median | Measure the frequency of the third formant. |
| f4_median | Measure the frequency of the fourth formant. |

Table 2: **Description of spectral features.**

| **Feature** | **Description** |
| --- | --- |
| chroma_stft | Chromogram from a waveform or power spectrogram. |
| spectral centroid | Compute the spectral centroid. |
| spectral bandwidth | P'th-order spectral bandwidth. |
| spectral rolloff | Compute roll-off frequency. |
| chroma_cqt | Constant-Q chromagram. |
| chroma_cens | Chroma variant 'Chroma Energy Normalized'. |
| melspectrogram | Mel-scaled spectrogram. |
| mfcc | Mel-frequency cepstral coefficients. |
| spectral contrast | Compute spectral contrast. |
| poly features | Coefficients of fitting an nth-order polynomial to the columns of a spectrogram. |
| tonnetz | Tonal centroid features. |
| spectral flatness | Compute spectral flatness. |
