# Supplementary material for: Exploring Speech Biosignatures for Traumatic Brain Injury and Neurodegeneration: Pilot Machine Learning Study
Source: JMIR Neurotechnol. 2025 Feb 12;4:e64624. doi: 10.2196/64624 (PMC12671332; doi:10.2196/64624)
Supplement: Multimedia Appendix 2 [file neuro-v4-e64624-s002.docx]

$$\hat{q}_{i}=\sum_{d=1}^{D} f_{d}\left( p_{i} \right) (1)$$

$$F_{1}= \frac{2*precision*recall}{precision+recall} (2)$$

Here:

- **Precision** measures the proportion of correctly predicted positive cases to the total number of predicted positive cases (true positives + false positives). It evaluates the ability of the model to suppress false positives.
- **Recall** (or sensitivity) measures the proportion of correctly predicted positive cases to the total number of actual positive cases (true positives + false negatives). It evaluates the model's ability to capture all relevant cases.
