# Supplementary material for: Exploring Speech Biosignatures for Traumatic Brain Injury and Neurodegeneration: Pilot Machine Learning Study
Source: JMIR Neurotechnol. 2025 Feb 12;4:e64624. doi: 10.2196/64624 (PMC12671332; doi:10.2196/64624)
Supplement: Multimedia Appendix 3 [file neuro-v4-e64624-s003.pdf]

Department of Computer Science and Engineering
University of Notre Dame

Informed Consent – Study of Impact of Neurological Conditions on Motor Function, Memory Function, Executive Function and Speech

What is the purpose of this project?
You are invited to participate in a research study about the impact of neurological conditions (including concussions, sleep disorders, stress, Alzheimer's Disease, Parkinson's Disease, etc.) on motor function, memory function, executive function, and speech. This study is being conducted Professors Christian Poellabauer and Sandra Schneider from the University of Notre Dame. The goal of this study is to collect motor function, memory function, executive function, and speech samples from subjects 18 years and older with or without known history of neurological and related conditions. Participation in this study is voluntary and non-participation will not affect any benefits, services, etc. received now or in the future.

If you agree to participate in this study, you will be asked to:

- Perform a simple set of motor, memory, executive function, and speech tasks where your computer, smartphone, or tablet will capture your speech, accelerometer data, gyroscope data, and screen interactions as they appear on the device. (The test takes about ten minutes). You will also answer a sequence of questions about your past/current health issues, where you can decide to skip all or some of the questions.

- Perform the same test again at regular intervals (e.g., once per month) or whenever changes in health have occurred.

In addition, you will be asked to answer brief surveys at the beginning and the end of the study. These surveys will ask for your age, gender, and pre-existing medical conditions, etc., that may have been experienced in the past. The health data provided by you as well as the recordings and information extracted from the recordings (e.g., pitch, word durations, pause durations, accelerometer data, gyroscope data, and screen interactions etc.) will be added to a repository for potential use in various research studies. Scientists at other institutions have to adhere to the same human subject protection requirements as the required by the University of Notre Dame to be allowed to access your data and speech recordings. No identifiable information (such as name, address, social security number, etc.) will be associated with your data/recordings!

Are there any risks in this research?
We don't believe this study will involve any risks for you. If you find some health questions upsetting, please tell us. You can choose to skip any question or stop the evaluation and reading test at any time. There may be unknown risks.

What are the benefits of being in this study?
Participation will not lead to any direct benefits. You may, as a result of participation, gain increased awareness of the potential long-term effects of neurological conditions and the use of novel tools to screen for such conditions.

How will we protect your privacy?
The surveys will ask for certain details of your medical history. We will protect your privacy to the extent allowed by law in several ways. First, all collected data will be identified only by a code number. Your name will be stored separately from your data and used only to track payments that we may make to you and ensure that future recordings you provide are linked to your initial recording. If you decide to participate, you will sign up for an account and only you will know your account name and password. All audio recordings and data will be saved electronically using state-of-the art security measures. The purpose of the study is to make the recordings and health data available to medical researchers to explore links between neurological conditions and speech. Therefore, the collected data will be shared among researchers at different institutions as long as the researchers comply with our protocols with regard to data/identity protection. Your agreement to participate in this study includes your agreement to this sharing of motor, memory, executive function and speech data collected from you. When such data is shared and when we report the results of this study, you will never be named or identified in any way.

We are required by law to make reports to prevent serious harm to you or others. If we see evidence of child abuse or neglect, we have to report it.

If you agree to participate, can you change your mind later?
Yes. You are free to join the study or decline. You can begin participating and then cancel your participation at any time, without any penalty, and any recordings and other data from your participation will be destroyed. Choosing not to participate in the study will not affect any services you may receive from the University of Notre Dame.

If you have any questions about this study, please contact Dr. Christian Poellabauer (574-631-9131, cpoellab@nd.edu) or Dr. Sandra Schneider (574-284-5268, sschneider@saintmarys.edu). If you have questions about your rights as a research

participant, please contact Notre Dame's Office of Research Compliance, (compliance@nd.edu), phone (574-631-1461).

Participant Certification:
I have read this form (or, it has been read to me), and I have had a chance to ask questions. My questions have been answered. I know that the data relating to my identity will be kept private, and that recordings and other data may be made available to other research groups under license.

I hereby agree to be part of this study. I know that I can drop out of the study at any time. I also agree to the use and sharing of my information as described above. By signing this, I verify that I am at least 18 years old. I have received a copy of this consent form to keep.
