# Supplementary material for: Exploring Speech Biosignatures for Traumatic Brain Injury and Neurodegeneration: Pilot Machine Learning Study
Source: JMIR Neurotechnol. 2025 Feb 12;4:e64624. doi: 10.2196/64624 (PMC12671332; doi:10.2196/64624)
Supplement: Multimedia Appendix 4 [file neuro-v4-e64624-s004.pdf]

Informed Consent Agreement

Study of Impact of TBI on Speech

What is the purpose of this project?

You are invited to participate in a research study about the impact of traumatic brain injuries (TBI) on human speech. This study is being conducted Professors Christian Poellabauer and Patrick Flynn from the University of Notre Dame, and Contect, Inc. The goal of this study is to collect speech samples from patients of all ages who may have experienced a mild or severe traumatic brain injury and therefore you are invited to participate in this study. Participation in this study is voluntary and non-participation will not affect any benefits, services, etc. received now or in the future.

If you agree to participate in this study, you will be asked to:

- Perform a simple reading test during an initial meeting (baseline testing) organized by Contect, Inc or your healthcare provider. In this test, you will be given a tablet device and you will be asked to read a sequence of words appearing on the screen of the tablet into the tablet's microphone.
- Perform the same reading several times afterwards, e.g., when meeting your healthcare provider.

Employees of Contect, Inc., researchers affiliated with Notre Dame and St. Mary's College and/or your healthcare provider will supervise this activity.

In addition, you may also be asked to answer brief surveys at the beginning and the end of the study. These surveys will ask for your age, gender, and pre-existing medical conditions such as concussions and TBI that may have been experienced in the past.
Note: the speech samples collected will only be used to study certain aspects of the speech of patients and will not be used to actually detect or diagnose TBI.

Are there any risks in this research?

We don't believe this study will involve any risks for you. If you find some health questions upsetting, please tell us. You can choose to skip any question. You are free to stop the assessment at any time. There may be unknown risks.

What are the benefits of being in this study?

Participation will not lead to any direct benefits. You may, as a result of participation, gain increased awareness of the potential long-term effects of undetected concussions and TBI.

How will we protect your privacy?

The surveys will ask for certain details of your medical history. We will protect your privacy to the extent allowed by law in several ways. First, all collected data will be identified only by a code number. Only the staff at the medical facility performing the testing and/or staff at Contect, Inc. will have your names and contact information. Audio recordings will be saved electronically using state-of-the art security measures. When we report the results of this study, you will never be named or identified in any way.

We are required by law to make reports to prevent serious harm to you or others. If we see evidence of child abuse or neglect, we have to report it.

If you agree to participate, can you change your mind later?

Yes. You are free to join the study or decline. If you join and later change your mind, you can withdraw from the study at any point, without any penalty, and audio recordings from your participation will be destroyed immediately. Choosing not to join the study will not affect any services you may receive from the University of Notre Dame, St. Mary's College, or your school or employer.

If you have any questions about this study, please contact Dr. Christian Poellabauer (574-631-9131, cpoellab@nd.edu) or Dr. Patrick Flynn (574-631-8803, flynn@nd.edu). If you have questions about your rights as a research participant, please contact Notre Dame's Office of Research Compliance, (compliance@nd.edu), phone (574-631-1461).

Participant Certification:

I have read this form (or, it has been read to me), and I have had a chance to ask questions. My questions have been answered. I know that the data on me will be kept private.

I hereby agree to be part of this study. I know that I can drop out of the study at any time. I also agree to the use and sharing of my information as described above. By signing this, I verify that I am at least 18 years old. I have received a copy of this consent form to keep.

________________________________ ____________________

Name Birth Date

________________________________ ____________________

Signature Date Signed

Current Address ____

Current Phone Number ____________________________________
